# Functional role and ribosomal position of the unique N-terminal region of DHX29, a factor required for initiation on structured mammalian mRNAs

**DOI:** 10.1093/nar/gkab1192

**Published:** 2021-12-09

**Authors:** Trevor R Sweeney, Vidya Dhote, Ewelina Guca, Christopher U T Hellen, Yaser Hashem, Tatyana V Pestova

**Affiliations:** Department of Cell Biology, SUNY Downstate Health Sciences University, 450 Clarkson Avenue, MSC 44, Brooklyn, NY 11203, USA; The Pirbright Institute, Woking, Surrey, UK; Department of Cell Biology, SUNY Downstate Health Sciences University, 450 Clarkson Avenue, MSC 44, Brooklyn, NY 11203, USA; INSERM U1212 Acides nucléiques : Régulations Naturelle et Artificielle (ARNA), Institut Européen de Chimie et Biologie, Université de Bordeaux, Pessac 33607, France; Department of Cell Biology, SUNY Downstate Health Sciences University, 450 Clarkson Avenue, MSC 44, Brooklyn, NY 11203, USA; INSERM U1212 Acides nucléiques : Régulations Naturelle et Artificielle (ARNA), Institut Européen de Chimie et Biologie, Université de Bordeaux, Pessac 33607, France; Department of Cell Biology, SUNY Downstate Health Sciences University, 450 Clarkson Avenue, MSC 44, Brooklyn, NY 11203, USA

## Abstract

Translation initiation on structured mammalian mRNAs requires DHX29, a DExH protein that comprises a unique 534-aa-long N-terminal region (NTR) and a common catalytic DExH core. DHX29 binds to 40S subunits and possesses 40S-stimulated NTPase activity essential for its function. In the cryo-EM structure of DHX29-bound 43S preinitiation complexes, the main DHX29 density resides around the tip of helix 16 of 18S rRNA, from which it extends through a linker to the subunit interface forming an intersubunit domain next to the eIF1A binding site. Although a DExH core model can be fitted to the main density, the correlation between the remaining density and the NTR is unknown. Here, we present a model of 40S-bound DHX29, supported by directed hydroxyl radical cleavage data, showing that the intersubunit domain comprises a dsRNA-binding domain (dsRBD, aa 377–448) whereas linker corresponds to the long α-helix (aa 460–512) that follows the dsRBD. We also demonstrate that the N-terminal α-helix and the following UBA-like domain form a four-helix bundle (aa 90–166) that constitutes a previously unassigned section of the main density and resides between DHX29’s C-terminal α-helix and the linker. In vitro reconstitution experiments revealed the critical and specific roles of these NTR elements for DHX29’s function.

## INTRODUCTION

Translation initiation on the majority of eukaryotic mRNAs occurs by the scanning mechanism (1). The process starts with formation of 43S preinitiation complexes (43S PICs) comprising 40S ribosomal subunits, Met-tRNA_i_^Met^/eIF2/GTP, and eukaryotic initiation factors (eIFs) 3, 1 and 1A. 43S PICs first attach to the cap-proximal region of mRNA in a process that is mediated by eIF4A, eIF4B and eIF4F that cooperatively unwind the cap-proximal region, and then scan to the initiation codon where they stop and form 48S initiation complexes with established codon-anticodon base-pairing. eIFs 4A/4B/4F also assist 43S PICs during scanning. However, scanning through stable secondary structures additionally requires the DExH protein DHX29 (2). DHX29 is also essential for initiation on several viral mRNAs (3,4). Silencing of DHX29 inhibits general translation, causing disruption of polysomes and accumulation of mRNA-free 80S ribosomes, and suppresses cancer proliferation (5). It has more recently been reported that DHX29 also enhances antiviral innate immunity. Thus, DHX29 cooperates with the cytosolic nucleic acid detectors retinoic acid-inducible gene 1 (RIG-I) and the melanoma differentiation-associated protein 5 (MDA5) to recognize relatively short viral double-stranded (ds) RNA (<1 kb) and long dsRNA, respectively, and to trigger activation of innate immune responses and initiation of the adaptive immune response (6,7).

DHX29 belongs to the superfamily two DExH RNA helicases. It consists of a unique 534aa-long N-terminal region (NTR), and a common C-terminal DExH catalytic core comprising two consecutive RecA1/RecA2 domains followed by winged helix (WH), ratchet-like (RL) and DHX specific oligonucleotide/oligosaccharide-binding (OB) domains (8). DHX29 binds directly to 40S subunits and possesses ribosome-stimulated NTPase activity that is essential for its function in initiation (2,8,9,10). Ribosomal targeting elements of DHX29 were identified in both the common DExH core and the unique NTR (8,11).

In the absence of DHX29, intact stems can still enter the mRNA-binding cleft of scanning 43S PICs, but they cannot be threaded through its exit portion, rendering 48S complexes formed on AUGs downstream of such stems susceptible to dissociation by eIF1 (12). If 48S complexes form on AUGs immediately preceding intact stems, the stem and the region between the stem and the AUG codon accumulate in the A site, making such complexes susceptible to dissociation by DHX29 (12). When present at the beginning, DHX29 promotes unwinding of mRNA stable secondary structures and ensures its linear nucleotide-by-nucleotide inspection. Interestingly, DHX29 also prevents formation of aberrant 48S complexes characterized by the +8–9-nt toe-prints that were attributed to the incompletely closed conformation of 48S complexes that allows reverse transcriptase to penetrate further (12), suggesting that DHX29 promotes a more closed conformation of the mRNA binding channel that could also enhance codon-anticodon base-pairing. Consistently, DHX29 was found to reduce leaky scanning (13). However, individually DHX29 is not a processive RNA helicase (2), and the main question concerning the mechanism of its action, whether it promotes mRNA unwinding directly or acts by inducing cyclic conformational changes 43S PICs, remains unresolved.

The cryo-EM structure of the DHX29-bound 43S PICs (9,10) revealed that the main DHX29 mass is located around the tip of helix (h) 16 of 18S rRNA, where it bridges h16 with the beak by interacting with ribosomal proteins uS3, eS10 and eS12, and establishes extensive contact with the eIF3b–eIF3i–eIF3g module that resides at the mRNA entrance underneath h16. The main mass extends via a linker to the subunit interface where it forms a small domain next to the eIF1A binding site. This intersubunit domain interacts with ribosomal proteins eS30 and uS12, and weakly with h34, thereby bridging the body with the beak just outside the A site. Although the homology-based model of the DExH core can be fitted to the cryo-EM structure occupying a large part of the main DHX29 density located around the tip of h16 (9,10), the correspondence of other parts of the density to specific elements in the NTR and the identity of the intersubunit domain remained unknown.

The NTR is required for DHX29’s ribosome-dependent NTPase activity and for its function in 48S complex formation (8). We have previously found that in its C-terminal half, the NTR contains a highly conserved dsRNA-binding domain (dsRBD, aa 377–448) that is critical for DHX29’s function (8). Thus, replacing the dsRBD by Ala spacers of various lengths nearly abrogated ribosomal association and 40S-stimulated NTPase activity of DHX29 without affecting its general ssRNA-dependent NTPase activity, indicating the dsRBD’s essential role in ribosomal association (8). Consistently, dsRBD deletion mutants were also inactive in 48S complex formation on structured mRNAs (8). dsRBDs generally display a large degree of diversity while maintaining the overall α_1_β_1_β_2_β_3_α_2_ fold (14). dsRBDs contain three regions that are typically involved in RNA binding: region 1 is in helix α_1_, region 2 is in the loop between the first and second β-strands and region 3 is at the beginning of helix α_2_ (14). The DHX29 dsRBD lacks motifs associated with RNA binding such as conserved positively charged amino acids at the beginning of helix α_1_, suggesting the non-canonical interaction between the dsRBD and the 40S subunit. However, although essential, the dsRBD constitutes only a small part of the long unique conserved DHX29’s NTR. To dissect the role of the NTR in DHX29’s function, we identified all its other structural elements, modeled their ribosomal positions, and assayed their individual contributions to DHX29-mediated 48S complex formation on structured mRNAs.

## MATERIALS AND METHODS

### Plasmids

Expression vectors for His_6_-tagged eIF1 and eIF1A (15), eIF4A and eIF4B (16), eIF4G_736-1115_ (17) and *Escherichia coli* methionyl tRNA synthetase (18), and the transcription vectors for (AUG at -6)-Stem mRNA (12) and tRNA_i_^Met^ (19) have been described. The vector for expression of the N-terminally FLAG-tagged and C-terminally His_6_-tagged *wt* DHX29 contained the DHX29 sequence, which had been optimized by selection of synonymous codons to ensure correct co-translational folding in *E. coli* (DAPCEL, Inc., Cleveland, OH) (3), cloned into pET16b between NcoI and BamHI restriction sites (8). This vector was used to generate all full-length DHX29 point and deletion/insertion mutants used in this study by NorClone Biotech Laboratories (London, Ontario, Canada). The vector for expression of DHX29(1–534) has been described (8). Expression vectors for N-terminally truncated DHX29 and for the corresponding N-terminal fragments were generated in-house by PCR mutagenesis. Vectors for expression of N-terminally truncated DHX29 fragments starting from amino acid residues 85, 111, 219, 261, 331, 346 or 376 and with N-terminal FLAG and C-terminal His_6_ tags were generated by cloning DNA between NcoI and RsrII sites of pET16b. Vectors for expression of N-terminal DHX29(1–464) and DHX29(1–519) fragments and N-terminally truncated DHX29(475–1369), DHX29(522–1369) and DHX29(535–1369) fragments, in all instances with N-terminal FLAG and C-terminal His_6_ tags, were generated by cloning DNA between NcoI and XhoI sites of pET28a. Vectors for expression of N-terminal DHX29(1–84) and DHX29(1–328) fragments with an N-terminal His_6_ tag followed by a thrombin cleavage site and a T7 tag were made by cloning DNA between BamHI and XhoI sites of pET28a. mRNA and tRNA_i_^Met^ were transcribed using T7 RNA polymerase, and *in vitro* transcribed tRNA_i_^Met^ was aminoacylated using recombinant *E. coli* methionyl tRNA synthetase as described (18).

### Purification of ribosomal subunits and initiation factors

Native ribosomal 40S subunits, eIF2 and eIF3 were purified from RRL (Green Hectares, Oregon, WI), and recombinant eIF1, eIF1A, eIF4A, eIF4B, eIF4G_736–1115_ and *E. coli* methionyl tRNA synthetase were expressed and purified from *E. coli* BL21(DE3) (20). Recombinant *wt* and mutant forms of DHX29 were expressed in 4–12 liters of *E. coli* BL21 Star (DE3) after induction with 0.5 mM IPTG overnight at 16°C. Full-length *wt* and mutant forms of DHX29 were purified by Ni-NTA affinity chromatography followed by FPLC on MonoS HR5/5 column (8). N-terminal DHX29(1–464) and DHX29(1–519) fragments and N-terminally truncated DHX29(475–1369), DHX29(522–1369) and DHX29(535–1369) fragments were isolated by FLAG affinity chromatography (Sigma-Aldrich) following Ni-NTA affinity purification. N-terminal DHX29(1–84) and DHX29(1–328) fragments were isolated by Ni-NTA affinity purification, treated with thrombin overnight at 4°C and passed over Ni-NTA beads again to remove impurities.

### Directed hydroxyl radical cleavage

DHX29 Cys mutant proteins were derivatized with Fe(II)-BABE by incubating 30 μg DHX29 with 1 mM Fe(II)-BABE in 100 μl buffer A (80 mM HEPES pH 7.5, 300 mM KAc and 10% glycerol) for 30 min at 37°C as described (21). Derivatized proteins were separated from unincorporated reagent by buffer exchange on Microcon YM-30 filter units and stored at −80°C. 40S ribosomal complexes containing [Fe(II)-BABE]-DHX29 were formed by incubating 10 pmol 40S subunits, 15 pmol eIF3, 12 pmol U_70_ and 10 pmol [Fe(II)-BABE]-DHX29 in 50 μl buffer B (20 mM HEPES (pH 7.5), 100 mM KCl, 2.5 mM MgCl_2_ and 5% glycerol) for 10 min at 37°C. To generate hydroxyl radicals, reaction mixtures were supplemented with 0.025% H_2_O_2_ and 5 mM ascorbic acid and incubated on ice for 10 min. Reactions were quenched by adding 20 mM thiourea. Ribosomal RNA was phenol-extracted and analyzed by primer extension using avian myeloblastosis virus reverse transcriptase (AMV RT) (Promega) and [^32^P]-labeled primers complementary to different regions of 18S rRNA. cDNA products were resolved in 6% or 10% polyacrylamide sequencing gels. All experiments were repeated two to three times.

### Toe-printing analysis of 48S complex formation

48S complexes were assembled by incubating 1 pmol of (AUG at –6)-Stem mRNA (12) with 2 pmol 40S subunits, 4 pmol eIF2, 5 pmol Met-tRNA_i_^Met^, 3 pmol eIF3, 8 pmol eIF4A, 2 pmol eIF4B, 4 pmol eIF4G_736–1115_, 10 pmol eIF1, 10 pmol eIF1A in the presence or absence of 0.4 pmol of the indicated DHX29 mutants/fragments in 40 μl buffer C (20 mM Tris (pH 7.5), 100 mM KCl, 2.5 mM MgCl_2_, 2 mM DTT and 0.25 mM spermidine) supplemented with 1 mM ATP and 0.4 mM GTP for 10 min at 37°C. Ribosomal complexes were analyzed by toe-printing (20) using AMV RT and a [^32^P]-labeled primer complementary to the coding region of the mRNA. cDNA products were resolved in 6% polyacrylamide sequencing gels. All experiments were repeated at least three times. In experiments presented in Figure 4, the intensities of bands corresponding to assembled 48S complexes were quantified using ImageQuant.

### NTPase assay

The NTPase activity of DHX29 was assayed using [α-^32^P]CTP essentially as described (8). 0.15 pmol of *wt* DHX29 or combinations of N and C terminal fragments of DHX29 were incubated with 6.7 μM [α-^32^P]CTP in the presence or absence of 2 pmol 40S subunits or 20 pmol U_70_ RNA in a 10 μl reaction mixture containing buffer B at 37°C for 15 min. 2 μl aliquots were spotted onto polyethyleneimine cellulose plates for thin layer chromatography done using 0.8 M LiCl/0.8 M acetic acid. The NTPase activity was determined by formation of [α-^32^P]CDP. All experiments were repeated at least two to three times.

### Ribosomal association of DHX29 fragments

To investigate ribosomal binding of DHX29 fragments, 60 pmol DHX29 fragments were incubated with 30 pmol 40S subunits in 200 μl reaction mixtures containing buffer C supplemented with 1mM ATP and 0.4 mM GTP at 37°C for 10 min. The reaction mixtures then were subjected to centrifugation through a 10–30% (w/w) sucrose density gradients prepared in buffer C in a Beckman SW55 rotor at 53 000 rpm for 1 h 15 min. Fractions that corresponded to the 40S subunit peak were analyzed by SDS/PAGE with subsequent western blotting using FLAG-tag (Sigma-Aldrich) antibodies. These experiments were repeated three times.

### Modeling of the DHX29 NTR

A first model of the full-length human DHX29 was generated by AlphaFold (22) and retrieved from the AlphaFold Protein Structure Database (https://alphafold.ebi.ac.uk). The low-confidence predicted regions corresponding to unstructured coils (residues 1–80 and 170–375) were removed from the model before its analysis. The remaining model was rigid-body fitted into the cryo-EM reconstruction of the 43S PIC (10) using Chimera UCSF (23). To improve the fitting locally, the first α-helix and the UBA-like domain (residues 90–166) were rigid-body fitted as one module into their densities, resulting in very minor angular adjustments. The dsRBD and the following linker helix (residues 365–512) were also rigid-body fitted as one module into their densities, resulting similarly in very minor angular adjustments to their initial position. Finally, the model of the 43S PIC contained the 40S subunit, the Met-tRNA_i_^Met^/eIF2/GTP ternary complex, the eIF3 octamer core and eIF3d that were extracted from PDB model 6YAM, and the eIF3 subunits b and i that were extracted from PDB model 5A5U. The composite model including DHX29 was visualized and rendered in ChimeraX (24). The composite model generated in this way was used solely to corroborate and visualize the results of the DHRC and functional *in vitro* reconstitution experiments.

## RESULTS

### Mapping the ribosomal position of the N-terminal region of DHX29 by the directed hydroxyl radical cleavage technique

**Figure 1.**
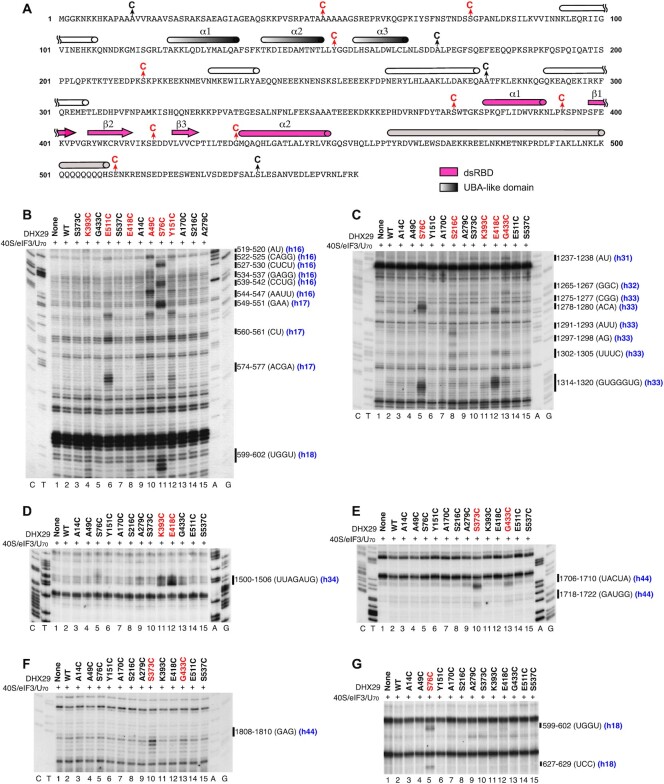
Directed hydroxyl radical cleavage of 18S rRNA in DHX29/40S/eIF3/U_70_ complexes from Fe(II) tethered to cysteines in the N-terminal region of DHX29. (**A**) Amino acid sequence of DHX29**’**s NTR showing predicted secondary structure elements (cylinders represent α-helices; arrows—β-strands). The UBA-like and dsRBD domains are colored grey and pink, respectively. The helical region downstream of the dsRBD is in light brown. The positions of Cys residues introduced into DHX29’s NTR are indicated. Cysteines, from which hydroxyl radical cleaved 18S rRNA, are in red. (**B–G**) Analysis of directed hydroxyl radical cleavage of 18S rRNA in DHX29/40S/eIF3/U_70_ complexes. Sites of cleavage were mapped by primer extension inhibition. Cysteines, from which hydroxyl radicals cleaved 18S rRNA, are in red. Positions of cleaved nucleotides are shown on the right. Lanes G, A, T, C depict 18S rRNA sequence generated from the same primer.

The Phyre2 (25) structural prediction of the N-terminal region of DHX29 predicted the presence of an ubiquitin-associated-like (UBA-like) domain (aa 121–164) (as previously suggested, 26) and the previously identified double-stranded RNA-binding domain (dsRBD, aa 377–448; 8) followed by a long α-helix containing a kink caused by the conserved P_487_ and a polyglutamine stretch Q_501–508_ (Figure 1A). The rest of the region was predicted to be largely unstructured, with sparse α-helices. The degree of DHX29 amino acid conservation correlated with identified structural elements, being the highest in the UBA-like and dsRBD domains and their immediately adjacent regions (Supplementary Figure S1).

To determine the overall ribosomal position of the DHX29-NTR, we employed the directed hydroxyl radical cleavage (DHRC) technique (27), which has previously been used to identify ribosomal positions of other eIFs (e.g. 28,29,30). In this approach, locally generated hydroxyl radicals cleave 18S rRNA in the vicinity of Fe(II) tethered to cysteine residues on the surface of the factor via the linker 1-(*p*-bromoacetamidobenzyl)–EDTA. Sites of cleavage are then determined by primer extension. Thirteen cysteines were introduced individually throughout the entire DHX29-NTR (Figure 1A). All DHX29 Cys mutants were active in 48S complex formation on structured mRNA (Supplementary Figure S2). We have previously found that DHX29 binds stably to ribosomal complexes containing 40S subunits, eIF3 and polyU RNA (2). The ribosomal position of the DHX29-NTR was therefore determined using DHX29/40S/eIF3/U_70_ complexes. Distinct cleavages were obtained from 9 positions (shown in red in Figure 1A).

**Figure 2.**
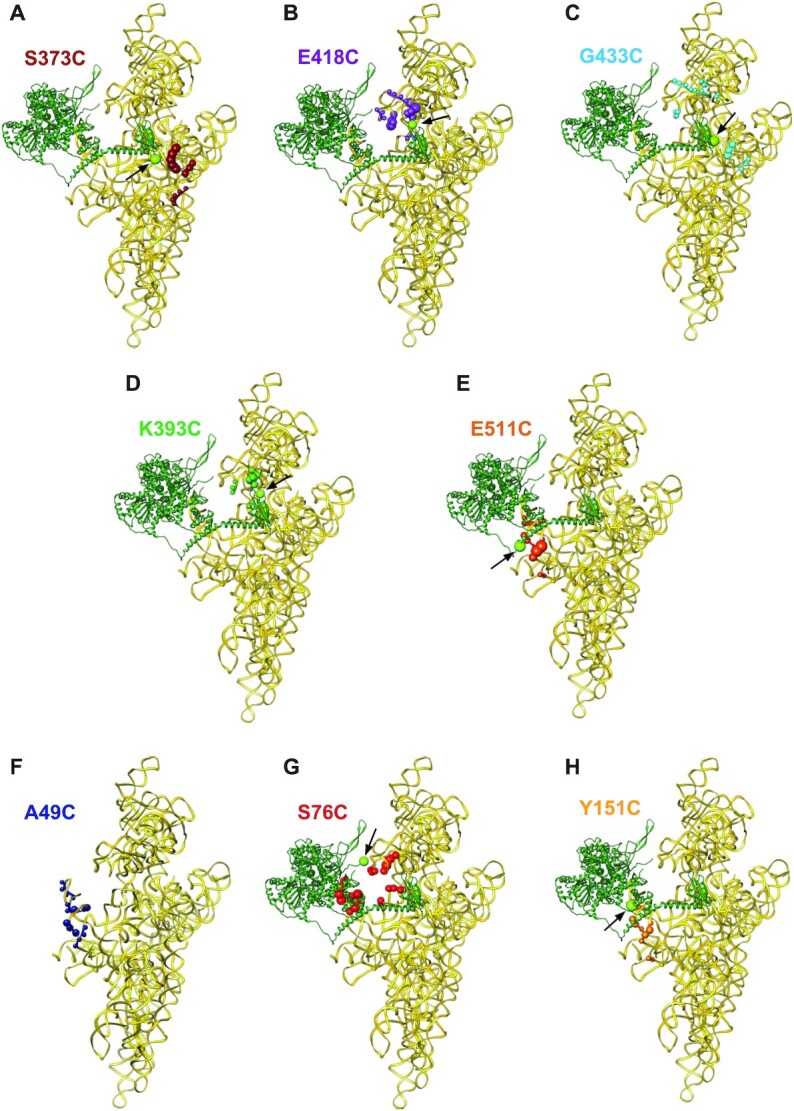
Positions of directed hydroxyl radical cleavage from Fe(II) tethered to cysteines in the NTR of DHX29 mapped onto corresponding regions of 18S rRNA. (A–H) Sites of cleavage (colored spheres) from (**A**) C373 immediately preceding the dsRBD, (**B–D**) C393, C418 and C433 within the dsRBD, (**E**) C511 immediately following the Q_501-508_ stretch, (**F, G**) C49 and C76 upstream of the UBA-like domain, and (**H**) C151 within the UBA-like domain mapped onto the structure of 18S rRNA (light-yellow ribbon, PDB code 4KZZ). The radius of the spheres is proportional to the strength of cleavage. In each panel the positions of mutated Cys residues in DHX29 modeled as in Figure 3 (forest green ribbon) are shown as chartreuse spheres and indicated by black arrows.

In the case of the dsRBD and the downstream region, cleavages occurred from C373 immediately preceding the dsRBD, C393, C418 and C433 within the dsRBD, and C511 immediately following the Q_501–508_ stretch. Hydroxyl radicals generated from C393 located in the loop between the first α-helix and the first strand of the β-sheet of the dsRBD resulted in low-intensity cleavages at nt. 601–602 (h18) and 1316–1318 (h33), and medium intensity cleavage at nt. 1500–1506 (h34) (Figure 1B-D). Addition of C418 between the second and third β-strands in the dsRBD led to strong cleavages at nt. 1314–1318 (h33) and 1500–1506 (h34), and weak cleavages at nt. 1278–1280 (h33), 1302–1305 (h33) and 601–602 (h18) (Figure 1B-D). Finally, introduction of C433 at the beginning of the second α-helix in the dsRBD yielded weak cleavages at nt. 1237–1238 (h31), 1265–1267 (h32), 1291–1293 (h33), 1302–1305 (h33), 1314–1317 (h33), 1706–1708 (h44) and 1808–1810 (h44) (Figure 1C, E and F). Hydroxyl radicals generated from C373 immediately preceding the dsRBD induced cleavage in h44 (with stronger cuts at nt. 1706–1710, medium cuts at nt. 1808–1810 and weaker ones at nt. 1718–1722; Figure 1E, F), whereas hydroxyl radicals generated from C511 immediately following the Q_501-508_ stretch of consecutive Gln residues induced strong cleavage at nt. 574–577 and weaker cleavages at nt. 549–551 and 560–561 in h17, and weak cleavages at nt. 539–542 and 519–520 in h16 (Figure 1B). On the 40S subunit structure, all cleavages from cysteines in the predicted dsRBD (C373, C393, C418 and C433) (Figure 2A-D) clustered in the area surrounding the small intersubunit domain of DHX29, whereas cleavages from C511 (Figure 2E) were located at the junction of the main density and the linker connecting the main density with the intersubunit domain. These data are consistent with the dsRBD constituting the intersubunit domain and the long α-helix downstream of the dsRBD forming the linker.

In the case of the region upstream of the dsRBD, strong cleavages were observed from C49 and C76 preceding the predicted UBA-like domain, and C151 within this domain. Hydroxyl radicals generated from C49 induced cleavage of variable intensity in h16 (strong cleavage at nt. 539–542 and weaker cleavage at nt. 519–520 and 527–530) and in h17 (medium intensity cleavage at nt. 549–551 and 574–577) (Figure 1B). Introduction of C151 in the loop between the second and third helices of the predicted UBA-like domain resulted in a similar pattern of cleavages with slightly different intensities: low to medium intensity cleavages at nt. 519–520 and 539–540 in h16, and low to medium intensity cleavage at nt. 549–551, 560–561 and 574–577 in h17 (Figure 1B). Interestingly, cleavages from C49 and C151 were very similar to those obtained from C511 located immediately following the Q_501-508_ stretch. Strong cleavage also occurred from C76, but this pattern of cleavage differed from those obtained from C49, C151 and C511. Thus, hydroxyl radicals generated from C76 cleaved 18S rRNA at nt. 522–525, 534–537 and 544–547 in h16, nt. 599–602 and 627–629 in h18, and nt. 1275–1277 and 1317–1320 in h33 (Figure 1B, C and G). Very weak cleavages were also observed from C216 downstream of the UBA-like domain at nt. 1291–1293, 1297–1298 and 1302–1305 in h33 (Figure 1C). On the 40S subunit structure, cleavages from the region upstream and within the predicted UBA-like domain (Figure 2F–H) clustered in close proximity to the main density suggesting that this region may form part of it.

### Modeling the NTR of the ribosome bound DHX29

**Figure 3.**
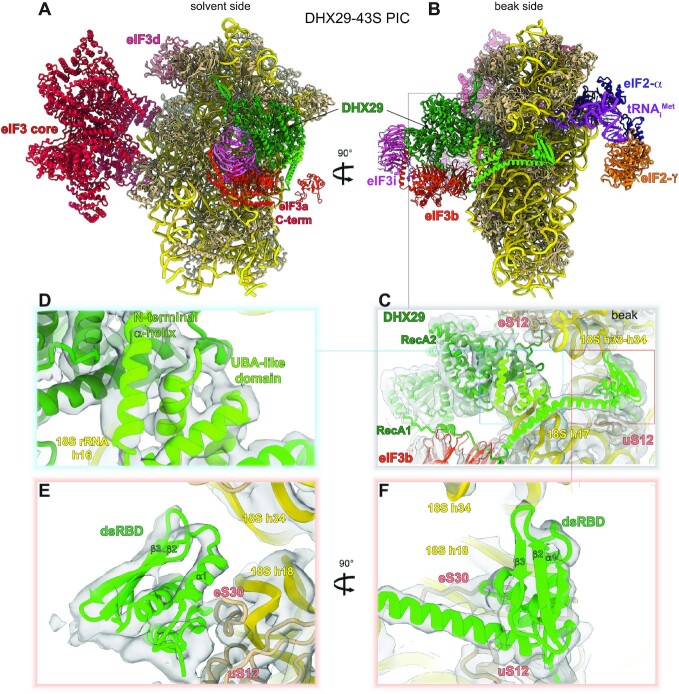
Model of DHX29 bound to the 43S pre-initiation complex. (A, B) The model of the DHX29-bound 43S pre-initiation complex (PDB code 6YAM) with the N-terminal region of DHX29 (AlphaFold model) and eIF3 peripheral subunits (PDB code 5A5U) seen from the solvent side (**A**) and beak side (**B**). (**C**) DHX29 atomic model displayed in its assigned electron density (10). Close-up view of (**D**) the N-terminal α-helix and the UBA-like domain and (**E**, **F**) the dsRBD domain and the linker helix with the possible contacts with 18S rRNA and ribosomal proteins.

To model the position of DHX29’s NTR on the 40S subunit, the structure of DHX29 was first modeled with AlphaFold (22). The resulting model was rigid-body fitted into the cryo-EM reconstruction of the mammalian DHX29-bound 43S PIC solved at an intermediate resolution (∼6Å) (10) (Figure 3). The AlphaFold model readily fits the DHX29 dependent density with only minor adjustments over some secondary structure elements applied to optimize the fit. As the presented model is predictive and validated by an intermediate-resolution cryo-EM structure, we restrict the description of the DHX29 structure to secondary-structure elements only.

As described above, the unique N-terminal region presents an unusual arrangement of domains that are atypical of known helicases (Supplementary Figure S3). The first ∼80 residues do not appear to form any specific structure and were modeled by AlphaFold as a long unstructured element, corroborated by the lack of clear attributable densities in the 43S PIC cryo-EM reconstruction. The first N-terminal α-helix and the following UBA-like domain comprising three short α-helices form a four-helix bundle (amino acids 90–166), which faces the rRNA h16 and is situated between the C-terminal α-helix of DHX29 and the linker helix following the dsRBD (Figure 3A–D and Supplementary Figure S3). The presence of the N-terminal α-helix and the UBA-like domain in this position allows bridging both ends of DHX29 and forms a solid tertiary structure that spans the 40S subunit from the mRNA channel entrance to the A-site. The UBA-like domain is followed by a long, mostly unstructured coil with sparse α-helices that spans from residue 166 to 375, up to the dsRBD, and lacks clear attributable densities in the 43S PIC cryo-EM reconstruction.

The dsRBD comprises the intersubunit domain of DHX29 and interacts with the 18S rRNA and two ribosomal proteins, eS30 and uS12 (Figure 3E, F and Supplementary Figure S3). Thus, the hairpin between β-strands 2 and 3 (residues 415–421) interacts with 18S rRNA h34, while the first α-helix (residues 380–390) interacts with the N-terminal tail of eS30 (between residues 75 and 82). Another interaction can be observed between the very last residues adjacent to the dsRBD (residues 375–379) and uS12 (a coil between residues 89 and 97). The following helical linker that extends from the dsRBD towards the main DHX29 density is ∼50 residues long (residues 460–512) and adopts a boomerang-like shape, as the helix is kinked at its middle (Figure 3E, F and Supplementary Figure S3). It interacts lightly with h17 of the 18S rRNA, where it ends with the Q_501-508_ stretch. The C-terminal end of this linker helix (from residue 512) extends as an unstructured coil to the RecA1 domain of DHX29 on the solvent side, facing h16 of the 18S rRNA, at the mRNA channel entrance (Figure 3A, B). Structurally, this linker helix connects the dsRBD located at the intersubunit side, near the A-site, to the solvent side main domain of DHX29 bound at h16.

The modeled ribosomal position and orientation of the structural elements in the DHX29’s NTR are in excellent agreement with the DHRC data. Thus, all Cys residues from which hydroxyl radical cleavages were obtained are in close proximity to the cleavage sites (Figure 2A–E and H), and the relative intensity of cleavage generally correlates with the distance between the Cys residues and the cleavage sites (Supplementary Table S1). Discrepancies between the distances and the cleavage intensity from Cys76 (Supplementary Table S1) may result from its position being only approximate because it lies at the extremity of the modeled region (Figure 2G). The distances for cleavages from Cys49 (Figure 2F) could not be analyzed because the corresponding N-terminal part of DHX29 is outside of the model. The lack of efficient hydroxyl radical cleavage from the region between the UBA-like domain and the dsRBD is consistent with the prediction of its largely disordered and potentially flexible nature, suggesting that it serves as a physical link between the dsRBD and the upstream structural unit.

### Functional analysis of the DHX29 linker region

**Figure 4.**
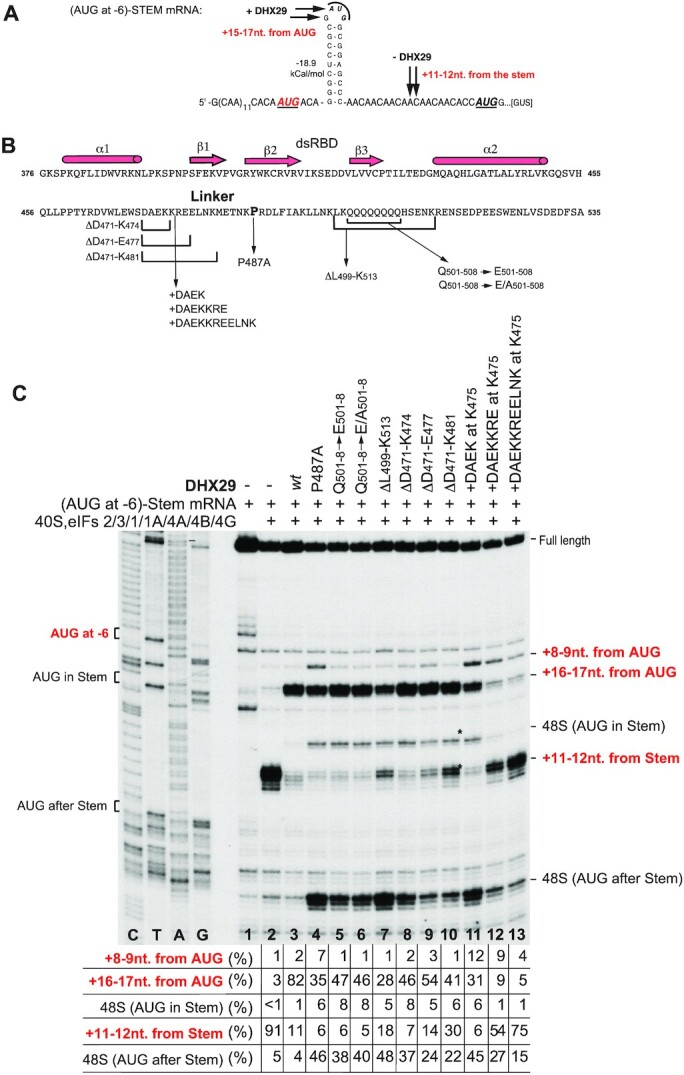
Mutation of the linker region disrupts the activity of DHX29 in 48S complex formation. (**A**) Sequence of the 5′ UTR of ‘(AUG at −6)-STEM’ mRNA, containing three AUG triplets: 6 nt before the stem, in the loop of the stem, and 21 nt downstream from the stem. Positions of toe-prints of 48S initiation complexes assembled with and without DHX29 are indicated by arrows. (**B**) Schematic representation of mutations introduced into the linker region connecting the dsRBD with the main density of DHX29. (**C**) Toe-printing analysis of the activity of DHX29 linker mutants in promoting 48S complex formation on (AUG at −6)-Stem mRNA. The positions of assembled ribosomal complexes and full-length cDNA are indicated on the right side of the panel. The positions of initiation codons are shown on the left. Lanes C/T/A/G depict the corresponding DNA sequence generated from the same primer. In each case, the relative efficiency of formation of different 48S complexes was quantified on the basis of three experiments with the combined efficiency of formation of complexes at all sites defined as 100%. Standard deviations (omitted for clarity) did not exceed 6.5%.

To investigate the functional roles of individual elements in the N-terminal region of DHX29, we applied the *in vitro* reconstitution approach. In this technique, 48S initiation complexes are assembled on mRNA from individual translation components (40S subunits, eIFs and Met-tRNA_i_^Met^), after which the position of 40S subunits is mapped by toe-printing. To assay the activity of DHX29, we employed (AUG at –6)-STEM mRNA containing a stable stem and three AUGs codons: at position −6 relative to the stem, in the loop of the stem, and downstream of the stem (Figure 4A; 12). In the absence of DHX29, 48S complexes form on the first AUG preceding the stem without its unwinding, and the stem is accommodated in the A site resulting in aberrant toe-prints + 11–12 nt downstream of the stem. DHX29 promotes unwinding of the stem and formation of 48S complexes with canonical toe-prints + 15–17 nt downstream from the first AUG. Unwinding of the stem also permits some leaky scanning resulting in low-efficiency 48S complex formation on AUGs within and downstream of the stem.

To examine the function of the linker that connects the dsRBD with the main density, we generated a panel of DHX29 mutants containing substitutions/deletions/insertions in the N-terminal part of the α-helix directly downstream of the dsRBD and in the Q_501–508_ stretch at the C-terminal end of this α-helix, as well as the substitution of the conserved Pro_487_ to Ala, which would affect the configuration of the linker helix (Figure 4B).

The substitution of the conserved Pro_487_ by Ala induced strong leaky scanning resulting in efficient assembly of 48S complexes at both downstream AUGs, and also led to the appearance of +8–9 nt. toe-prints that have previously been attributed to the incompletely closed conformation of 48S complexes that allow RT to penetrate further (12) (Figure 4C, lane 4). These data indicate the importance of the specific configuration of the linker.

Replacing all residues in the Q_501–508_ stretch by Glu or by an Ala/Glu mixture also increased leaky scanning, but it was less severe than in the case of the Pro487Ala substitution, and in contrast to this substitution, these mutations did not result in the appearance of +8–9 nt. toe-prints (Figure 4C, lanes 5–6). The effect of these substitutions is consistent with the weak interaction of this region with h17 of 18S rRNA that was noted above.

Next, we tested the effect of changing the length of the linker helix. To shorten it, we removed one, two or three turns N-terminal to P_487_ by deleting residues D_471_–K_474_, D_471_–E_477_ and D_471_–K_481_, respectively, or deleted the entire Q_501–508_ stretch with several flanking amino acids (DHX29_Δ499–513_) in the C-terminal part of the linker. To lengthen it, one, two or three turns were added at position K_475_ by inserting amino acids DAEK, DAEKKRE and DAEKKREELNK, respectively.

Shortening of the linker helix at the N-terminal part was tolerated relatively well, although in all cases we observed moderate leaky scanning and progressive strengthening of the aberrant +11–12 nt. toe-prints (Figure 4C, lanes 8–10). The Δ499–513 deletion at the C-terminal part of the linker had an effect analogous to that of the longest deletion in the N-terminal part, but with even stronger leaky scanning (Figure 4C, lane 7). A similar impairment of DHX29’s function was observed previously upon precise deletion of the Q_501–508_ stretch (11).

In contrast, lengthening of the linker helix had a much stronger influence on the activity of DHX29. Addition of one turn had an effect similar to that of the Pro487Ala substitution: strong leaky scanning and the appearance of the +8–9 nt toe-prints, indicating incomplete closure of 48S complexes (Figure 4C, lane 11). However, lengthening of the linker by two turns severely impaired the activity of DHX29 in 48S complex formation, whereas lengthening by three turns nearly abrogated its function (Figure 4C, lanes 12–13).

Thus, the specific configuration of the linker and a distinct structural rigidity of the connection between the dsRBD and the main helicase core are important for the activity of DHX29.

### Functional analysis of the DHX29 N-terminal region upstream of the dsRBD

**Figure 5.**
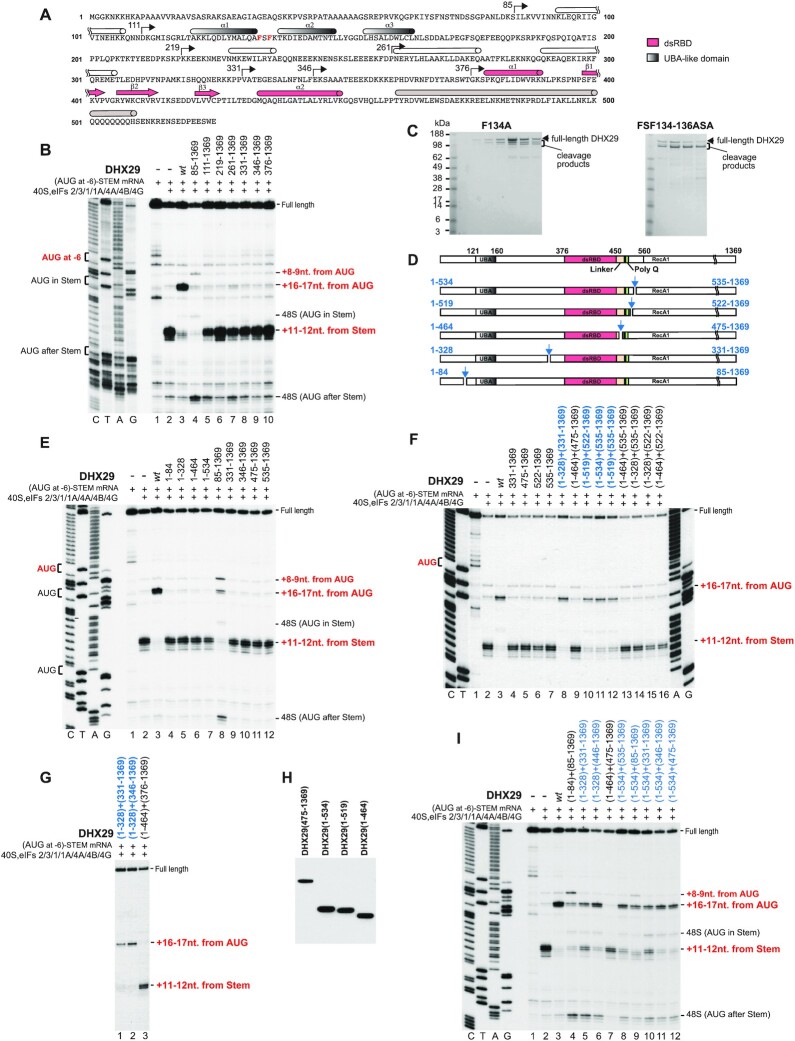
Activity of N-terminally truncated DHX29 in 48S complex formation is restored by addition of deleted regions *in trans* if the break is located upstream of the dsRBD or downstream of the Q_501-508_ stretch, but intactness of the linker region is required. (**A**) Amino acid sequence of DHX29**’**s NTR showing predicted secondary structure elements. The boundaries of N-terminal deletions in DHX29 are marked by arrows. Substituted hydrophobic amino acids in the UBA-like domain (F_134_ and F_136_) are in red. (**B**) Toe-printing analysis of the activity of the N-terminally truncated DHX29 mutants in promoting 48S complex formation on (AUG at −6)-Stem mRNA. (**C**) The integrity of purified F_134_/A_134_ and FSF_134-136_/ASA_134-136_ DHX29 mutants after elution from mono S assayed by SDS PAGE. (**D**) Schematic representation of the breaks introduced into DHX29’s NTR and of the resulting N and C terminal DHX29 fragments. The positions of the UBA-like domain, the dsRBD, the linker region and the Q_501-508_ stretch are indicated. (**E–G, I**) Restoration of the activity of the N-terminally truncated DHX29 mutants (panel D) in 48S complex formation on (AUG at −6)-Stem mRNA by addition *in trans* of the deleted regions, assayed by toe-printing. (**B, E–G, I**) The positions of assembled ribosomal complexes and full-length cDNA are indicated on the right side of each panel. The positions of initiation codons are shown on the left. Lanes C/T/A/G depict the corresponding DNA sequence generated from the same primer. (**H**) Association of DHX29 fragments with 40S subunits assayed by sucrose density gradient centrifugation followed by western blotting using anti-FLAG tag antibodies.

To evaluate the functional contribution of the region upstream of the dsRBD to the activity of DHX29 in 48S complex formation, we generated a panel of N-terminally truncated mutants guided by the secondary structure prediction (start sites of the mutants are shown in Figure 5A). It was previously reported that deletion of the first 54 amino acids did not affect the activity of DHX29 (11). However, removing the first 84 N-terminal amino acids strongly affected its function, leading to severe leaky scanning and the appearance of +8–9 nt. toe-prints indicating formation of incompletely closed 48S complexes. Although the first 54 residues of DHX29 are not very conserved, the 84aa-long N-terminal fragment contains a stretch of highly conserved amino acids preceding the first predicted α helix (Supplementary Figure S1), which could participate in the tertiary structure network that involves the N-terminal helix and the UBA-like domain and thus appears to be important for DHX29’s function. Additional deletion of the next 27 residues (containing the first N-terminal α-helix) completely abolished the activity of DHX29 (Figure 5B, lanes 4 and 5), which is consistent with the importance of this helix for the structural integrity of DHX29 suggested by the model (Figure 3).

UBA domains are formed by compact three-helix bundles with a large hydrophobic surface (comprising the loop 1 between α1 and α2, and the C-terminal part of α3) that interacts with ubiquitin (31,32). Similar hydrophobic surfaces occur in UBA domains that do not bind ubiquitin (33), but instead interact with other proteins (e.g. association of the UBA domain in the DNA repair protein HR23A with HIV-1 Vpr) (34). DHX29’s UBA domain also contains a predicted conserved hydrophobic surface, which includes two Phe residues (F134 and F136) in the loop between α1 and α2. Substitution of one (F_134_/A_134_), and particularly of both (FSF_134-136_/ASA_134-136_) phenylalanines increased proteolytic cleavage of DHX29 at specific positions in the N-terminus during its expression in *E. coli* (Figure 5C), suggesting that this area of the UBA-like domain might be involved in maintaining the structural integrity of DHX29. However, as stated above, our DHX29 model is predictive and was validated by an intermediate-resolution cryo-EM structure, and therefore does not allow us to comment with certainty on the region with which this hydrophobic surface might interact.

Interestingly, during purification of recombinant DHX29 containing N-terminal FLAG and C-terminal His_6_ tags sequentially over affinity columns for both tags, in addition to the full-length protein, the final product contained short (∼20 kDa) FLAG-tagged and long His-tagged DHX29 fragments. This suggested that the cleavage occurred in the N-terminal region and that the derived fragments were still interacting. The size of the N-terminal cleavage products implied that they included the UBA-like domain. The continued interaction of this N-terminal region with the rest of DHX29 is also consistent with our model in which this region is in intimate contact with the C-terminal region of DHX29. Association of the N-terminal cleavage fragments with the rest of the protein during DHX29 purification prompted us to investigate whether the activity of the N-terminally truncated mutants could be restored by addition *in trans* of the deleted regions.

Guided by the structural predictions, we introduced breaks at several positions: before the first α helix, in the region separating the last predicted α-helix and the dsRBD, in the linker helix, and after the Q_501-508_ stretch (Figure 5D). Individually, neither the N-, nor the C-terminal DHX29 fragments, except for the aforementioned DHX29_85-1369_ mutant, showed any activity in 48S complex formation (Figure 5E). Remarkably, the activity of N-terminally truncated DHX29 mutants was restored by the addition of deleted regions if the break was located in the region separating the last α helix and the dsRBD (DHX29_1–328_ + DHX29_331–1369_) (Figure 5F, lane 8) or after the Q_501-508_ stretch (DHX29_1–519_ + DHX29_522–1369_ and DHX29_1–534_ + DHX29_535–1369_) (Figure 5F, lanes 10 and 11). DHX29_1–328_ also complemented DHX29_346–1369_, but not DHX29_376–1369_ (Figure 5G), implying that aa 329–344 are dispensable, whereas the ∼30 aa preceding the dsRBD are required for DHX29’s function. In turn, DHX29_1–519_ restored the activity of DHX29_535–1369_ (Figure 5F, lane 12), indicating that aa 520–534 are not essential.

In contrast, no restoration occurred when the break was located within the linker helix (DHX29_1–464_ + DHX29_475–1369_) (Figure 5F, lane 9). Both fragments retained the ability to associate with 40S subunits (Figure 5H) indicating that the lack of their activity in 48S complex formation was not due to loss of ribosomal binding.

Strikingly, the DHX29_1–534_ fragment comprising the entire N-terminal region was able to restore the activities of DHX29_331–1369_, DHX29_346–1369_ as well as DHX29_475–1369_ (Figure 5I, lanes 10–12). It also to some extent complemented the activity of DHX29_85–1369_, which was not complemented by the corresponding aa 1–84 deleted region (Figure 5I, compare lanes 4 and 9). In the case of DHX29_1–534_ + DHX29_331–1369_ and DHX29_1–534_ + DHX29_346–1369_ complementation, the paired fragments contain a common region that included the dsRBD, linker helix and the following ∼25 aa. This raises the question of which fragment contributed the dsRBD and linker helix that functioned in 48S complex formation. Since DHX29 was able to tolerate the breaks upstream of the dsRBD as well as downstream of the Q_501-508_ stretch, DHX29_1–534_ could potentially function by both mechanisms: by providing only the region upstream of the dsRBD or by also displacing the dsRBD that is a part of the C-terminal fragment from the 40S subunit.

The ribosome-dependent NTPase activity of DHX29 is a prerequisite for its function in initiation on structured 5’UTRs (2,8). We therefore investigated the correlation between the NTPase activity and the activity in 48S complex formation of N-terminally truncated DHX29 mutants, depending on the presence *in trans* of the deleted regions. Since DHX29 lacks nucleotide specificity and can hydrolyze all four NTPs (2), the NTPase activity of DHX29 mutants was monitored using CTP because the protein preparations were potentially more likely to contain contaminants that could elevate the background level of ATP hydrolysis (8).

**Figure 6.**
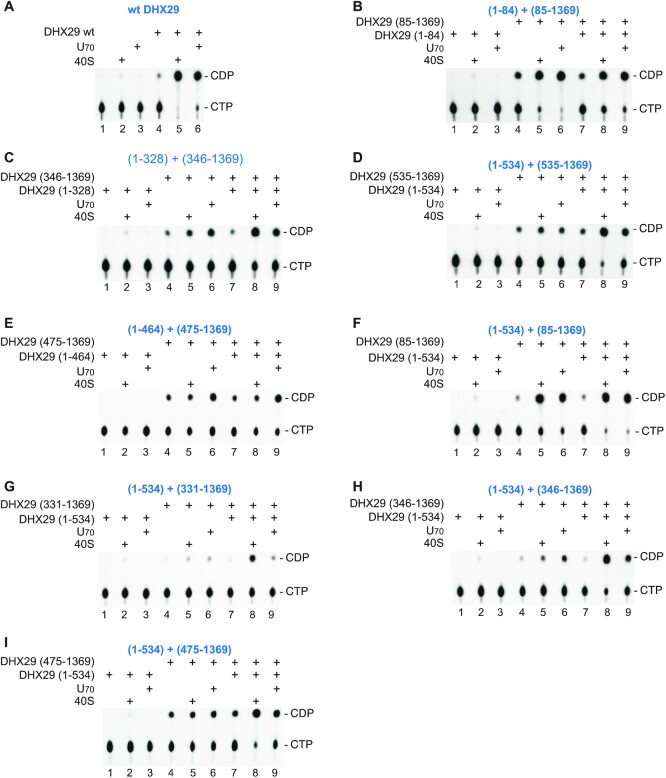
The NTPase activity of the N-terminally truncated DHX29 mutants depending on addition *in trans* of the deleted regions. (**A–I**) TLC analysis of the NTPase activity of the *wt* and N-terminally truncated DHX29 (Figure 5D) with/without addition *in trans* of DHX29 fragments corresponding to the deleted regions in the presence/absence of 40S subunits or U_70_ RNA. The positions of [^32^P]CTP and [^32^P]CDP are indicated on the right.

Consistent with our previous report (8), the NTPase activity of the full-length DHX29 was strongly stimulated by 40S subunits and slightly less so by U_70_ oligoribonucleotide (Figure 6A). The NTPase activity of individual N-terminally truncated mutants correlated with their activity in 48S complex formation. Thus, substantial stimulation of the NTPase activity by 40S subunits was observed only in the case of DHX29_85–1369_, but in contrast to the full-length protein, CTP hydrolysis was still stimulated more strongly by U_70_ (Figure 6B, lanes 4–6), and this phenotype was not reversed by addition of the deleted region (Figure 6B, lanes 7–9). The ability of deleted regions to restore 40S-dependent NTPase activity of N-terminally truncated DHX29 mutants also correlated with their ability to restore the activity of these mutants in 48S complex formation: complementation in the NTPase assay was observed if the break was located before the dsRBD or after the Q_501-508_ stretch, but not within the linker helix (Figure 6C–E, compare lanes 5 and 8). Again, the DHX29_1–534_ fragment comprising the entire NTR restored the activity of all N-terminally truncated mutants (Figure 6F–I, compare lanes 5 and 8).

Taken together, these results indicate that the N-terminal region preceding the dsRBD and containing the first N-terminal α-helix and the UBA-like domain is essential for DHX29’s ribosome-dependent NTPase activity and its function in 48S complex formation. Furthermore, they emphasize the importance of the communication between the dsRBD and the main density provided by the linker helix for DHX29’s function, and highlight the domain organization of DHX29, demonstrating that a fully intact polypeptide is not a prerequisite for DHX29 activity, and the breaks introduced before the dsRBD or after the linker helix are tolerated.

## DISCUSSION

The cryo-EM structure of the DHX29-bound 43S PIC revealed that the main DHX29 mass, which resides around the tip of helix 16, extends through a linker to the subunit interface where it forms a small domain next to the eIF1A-binding site (9,10). Although the homology-based model of the conserved C-terminal DExH-core of DHX29 comprising RecA, WH, RL and OB domains could be fitted to the main density (9,10), the ribosomal position of its unique 534aa-long NTR, as well as the identity and functional role of the small intersubunit domain remained unknown. Here we present a model of the 40S-bound DHX29, supported by results of DHRC experiments, which revealed the ribosomal positions of the structural elements in DHX29’s NTR. Thus, the intersubunit domain is formed by the dsRBD (aa 377–448), whereas the boomerang-like shaped linker that extends from the intersubunit domain towards the main DHX29 density corresponds to the long α-helix (aa 460–512) that follows the dsRBD. The first N-terminal α-helix and the following UBA-like domain comprising three short α-helices form a four-helix bundle (aa 90–166) that constitutes a previously unassigned section of the main density as supported by modelling and DHRC experiments, faces the rRNA h16, and is flanked between the C-terminal α-helix of DHX29 and the linker helix following the dsRBD. Interestingly, the dsRBD interacts with the 40S subunit in a non-canonical manner. Thus, in the case of DHX29, the first dsRBD region that is typically involved in interaction with RNA corresponds to the hydrophilic face of the first α-helix (residues 279–290), which is in firm interaction with eS30, and not with the rRNA. Moreover, the second RNA binding site at the tip of the second α-helix and the hairpin between β-strands 1 and 2 (DHX29’s residues 432–434 and 404–406, respectively) is accessible and not involved in the interaction with any rRNA elements either. Interestingly, a few other DExH proteins contain one or two dsRBDs as accessory domains, including DHX30 (14), DHX9 (RNA helicase A) and its *Drosophila* homolog Maleless (MLE) (35,36) and the RNA-editing associated helicase 2 from kinetoplastid mitochondria (37), but in contrast to DHX29, they all contain typical conserved RNA-binding residues, and their functional importance relates primarily in their RNA-binding activity (35,38,39,40,41). In MLE, dsRBD2 binds with the C-terminal OB domain to the central DExH module, forming the entrance to the helicase channel, consistent with the influence of dsRBD2 on helicase activity (36).

Identification of DHX29’s NTR regions constituting the intersubunit domain, the linker and the part of the main density allowed the critical roles of these elements in DHX29’s function to be revealed. Functional studies indicate that all identified structural elements of DHX29’s NTR are essential for its activity. Thus, deletion of the dsRBD, removal of the first N-terminal α-helix, as well as breaking the integrity of the linker helix abrogated DHX29’s ribosome-induced NTPase activity and its function in 48S complex formation on structured mRNAs ((8) and the present study). Mutagenesis of the linker helix also revealed that the linker supports a tensile connection between the main density and the intersubunit domain, since its shortening by three helical turns was tolerated relatively well, whereas lengthening by as little as one turn strongly inhibited DHX29-mediated 48S complex assembly on structured mRNA. Thus, the linker's tension appears to be functionally critical and ensures that the correct distance is maintained between two points of the ribosomal contact of DHX29. While the DExH core makes a number of contacts with the 40S subunit and the eIF3b–eIF3i–eIF3g module, it interacts with a highly flexible ribosomal feature, h16. It is therefore possible that the second, firm point of contact between DHX29 and the intersubunit face is required to stabilize the ribosomal interaction of the DExH core thereby promoting the correct conformation required for induction of the NTPase activity. The DHX29 region that includes the N-terminal α-helix and the UBA-like domain constitutes parts of the main density and participates in the stabilization of the DExH core and its communication with the intersubunit domain. The stable interactions of these N-terminal structural elements with the DExH core also underlie their ability to function without being covalently linked.

Interestingly, the main defect in initiation associated with DHX29 NTR mutants that retained some ability to promote mRNA unwinding and formation of 48S complexes on structured mRNA was strong leaky scanning. The correct spatial arrangement of the main density and the intersubunit domain is therefore also a prerequisite for efficient codon-anticodon base-pairing. The lack thereof likely interferes with conformational changes and release of eIF1 that accompany establishment of the codon-anticodon interaction. This also poses the question of whether DHX29 remains associated with 48S complexes or dissociates upon AUG recognition.

The principal outstanding question concerning the mechanism of DHX29’s action is whether it unwinds mRNA directly, acts indirectly by inducing conformational changes at the mRNA entrance or a combination of the two. DHX29 is not a processive RNA helicase (2), mutations in its ratchet helix do not affect the activity of DHX29 in scanning (8), and the peculiar ribosomal arrangement of its domains readily challenges an exclusive helicase-only mechanism of action for this protein. The N-terminal dsRBD of DHX29 is firmly bound to the intersubunit face of the 40S, while the main domains at the C-terminal end are bound to the solvent side at a fairly flexible region where the mRNA enters the mRNA-binding channel. From analyzing the ribosomal position of DHX29, it is plausible to hypothesize that it could control the opening and closing of the mRNA binding channel through NTP hydrolysis enabling mRNA entry, whereas the dsRBD firmly fixed at the A site would aid the disentanglement of mRNA secondary structures and the full accommodation of mRNA into the mRNA-binding channel.

However, despite the attraction of the indirect model, the possibility that DHX29 unwinds mRNA directly cannot strictly be discounted, which is in theory not mutually exclusive with the indirect model of action. Interestingly, the ribosomal position of DHX29 at the mRNA entrance is similar to that of the DExH-box helicase Ski2 except for the difference in the orientation of RecA1 domains (42). Although Ski2 extracts mRNA from 80S ribosomal complexes rather than promotes its entry (43), it acts directly with the 3'-terminal mRNA nucleotides threaded into the Ski2 RNA channel (42). Further elucidation of the mechanism of DHX29’s function will require determination of the structure of 43S PICs associated with DHX29 in different nucleotide-bound states and of DHX29-bound 48S complexes.

## Supplementary Material

gkab1192_Supplemental_File
