# Supplementary material for: Functional role and ribosomal position of the unique N-terminal region of DHX29, a factor required for initiation on structured mammalian mRNAs
Source: Nucleic Acids Res. 2021 Dec 9;49(22):12955–69. doi: 10.1093/nar/gkab1192 (PMC8682770; doi:10.1093/nar/gkab1192)
Supplement: gkab1192_Supplemental_File [file gkab1192_supplemental_file.pdf]

Supplemental Information

Functional role and ribosomal position of the unique N-terminal region of DHX29, a factor required for initiation on structured mammalian mRNAs

Trevor R. Sweeney[1,3*‡], Vidya Dhote[1*], Ewelina Guca[2], Christopher U.T. Hellen[1], Yaser Hashem[2‡] and Tatyana V. Pestova[1*‡]

[1] Department of Cell Biology, SUNY Downstate Health Sciences University, 450 Clarkson Avenue, MSC 44, Brooklyn, NY 11203, USA

[2] INSERM U1212 Acides nucléiques : Régulations Naturelle et Artificielle (ARNA), Institut Européen de Chimie et Biologie, Université de Bordeaux, Pessac 33607, France

[3] The Pirbright Institute, Woking, Surrey, United Kingdom.

[*] These authors contributed equally

[‡] Corresponding authors: Tatyana V. Pestova – tatyana.pestova@downstate.edu
Trevor R. Sweeney – trevor.sweeney@pirbright.ac.uk
Yaser Hashem – yaser.hashem@inserm.fr

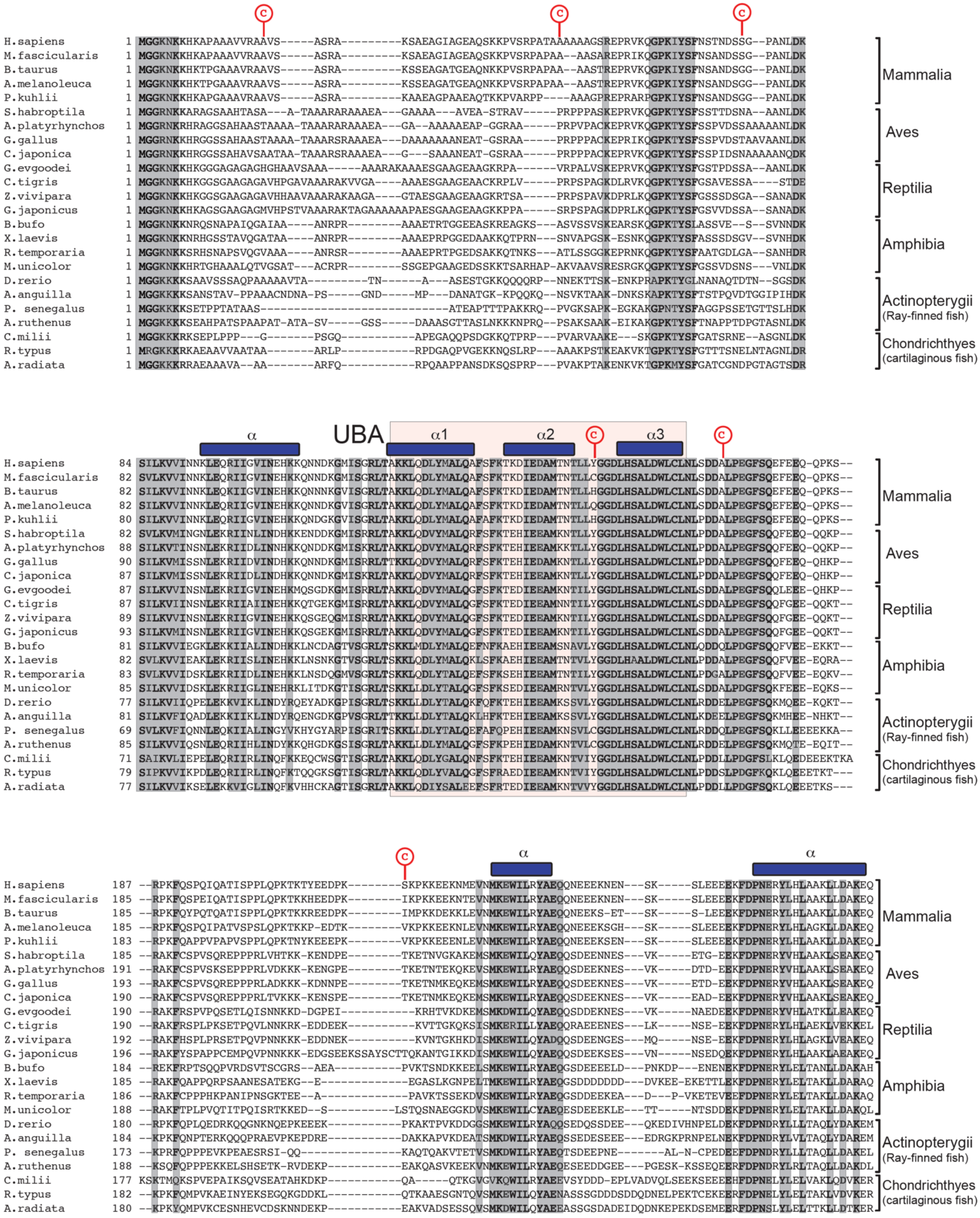
H.sapiens
M.fascicularis
B.taurus
A.melanoleuca
P.kuhlii
S.habroptila
A.platyrhynchos
G.gallus
C.japonica
G.evgoodei
C.tigris
Z.vivipara
G.japonicus
B.bufo
X.laevis
R.temporaria
M.unicolor
D.rerio
A.anguilla
P. senegalus
A.ruthenus
C.milii
R.typus
A.radiata
Mammalia
Aves
Reptilia
Amphibia
Actinopterygii
(Ray-finned fish)
Chondrichthyes
(cartilaginous fish)
UBA
α
α1
α2
α3
c

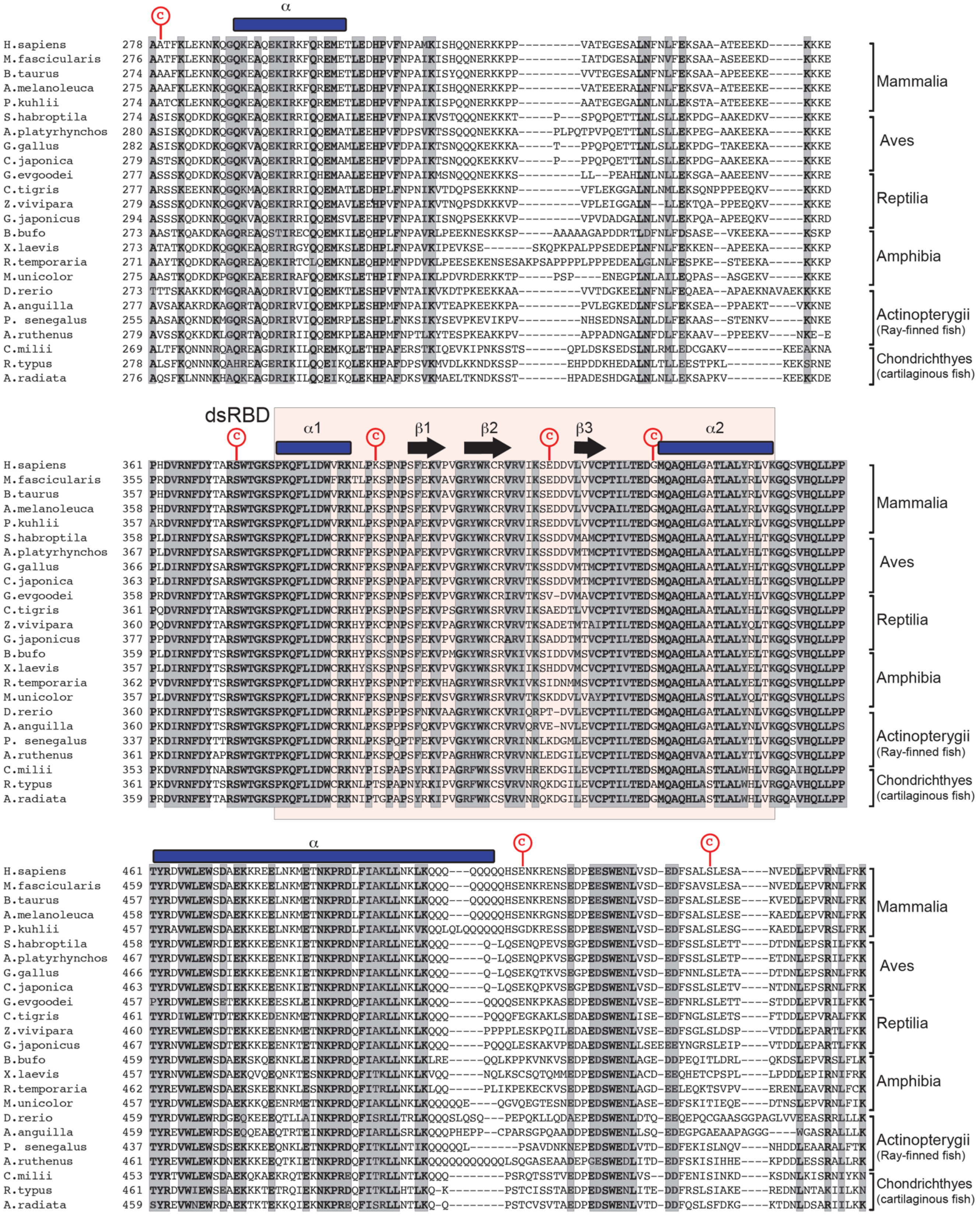

Figure S1. Alignment of the amino acid sequences of the N-terminal region of DHX29 from species of the phylum *Chordata*.

The GenBank accession numbers are for DHX29 from members of the classes *Mammalia*: *Homo sapiens* (NP_061903.2), *Macaca fascicularis* (crab-eating macaque) (EHH54259.1), *Bos taurus* (cattle)

(NP_001193063.1), *Ailuropoda melanoleuca* (giant panda) (XP_002928124.2), and *Pipistrellus kuhlii* (Kuhl's pipistrelle) (XP_036265063.1); *Aves* (birds): *Strigops habroptila* (Kakapo) (XP_030326824.1), *Anas platyrhynchos* (mallard) (XP_027302827.1), *Gallus gallus* (red junglefowl) (XP_015133056.2), *Coturnix japonica* (Japanese quail) (XP_015704195.1); *Reptilia*: *Gopherus evgoodei* (Goode's thornscrub tortoise) (XP_030422379.1, *Crotalus tigris* (tiger rattlesnake) (XP_039211600.1), *Zootoca vivipara* (common lizard) (XP_034956602.1), and *Gekko japonicus* (Schlegel's Japanese gecko) (XP_015278712.1); *Amphibia*: *Bufo bufo* (common toad) (XP_040277188.1), *Xenopus laevis* (African clawed frog) (NP_001091401.1), *Microcaecilia unicolor* (tiny cayenne caecilian) (XP_030048986.1), and *Rana temporaria* (common frog)(XP_040195378.1); *Actinopterygii* (Ray-finned fish): *Danio rerio* (zebrafish) (XP_697933.6), *Anguilla anguilla* (European eel) (XP_035246150.1), *Polypterus senegalus* (Senegal bichir) (XP_039606285.1) and *Acipenser ruthenus* (sterlet) (XP_033869089.2); and *Chondrichthyes* (cartilaginous fish): *Callorhinchus milii (*Australian ghostshark) (XP_007890373.1), *Rhincodon typus* (whale shark) (XP_020384667.1) and *Amblyraja radiata* (thorny skate) (XP_032886504.1).

Sequences were aligned using Clustal Omega. Residues that are identical or conserved in >90% of these sequences are shaded and are indicated in bold black or in grey font respectively. Predicted structural elements and domains are indicated.

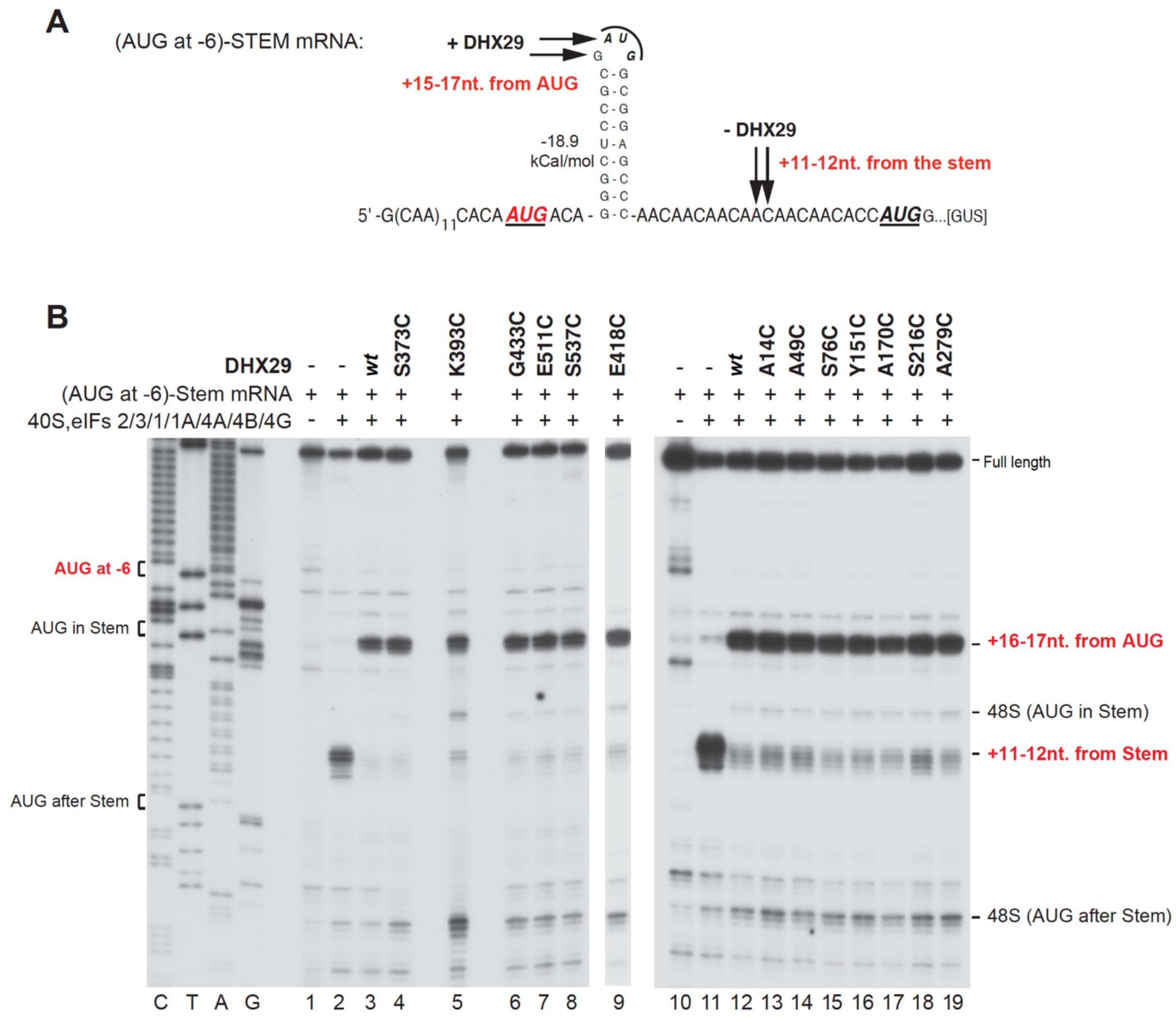

Figure S2. Activities of DHX29 Cys mutants in 48S complex formation on structured mRNA. (A) Sequence of the 5′ UTR of "(AUG at −6)-STEM" mRNA, containing three AUG triplets: 6 nt before the stem, in the loop of the stem, and 21 nt downstream from the stem. Positions of toe-prints of 48S initiation complexes assembled with and without DHX29 are indicated by arrows. (B) Toe-printing analysis of the activity of DHX29 linker mutants in promoting 48S complex formation on (AUG at −6)-Stem mRNA. The positions of assembled ribosomal complexes and full-length cDNA are indicated on the right side of the panel. The positions of initiation codons are shown on the left. Lanes C/T/A/G depict the corresponding DNA sequence generated from the same primer.

In the absence of DHX29, 48S complexes formed on the first AUG preceding the stem without its unwinding, and the stem was accommodated in the A site resulting in aberrant toe-prints +11-12 nt downstream of the stem. *wt* DHX29 and all its Cys mutants promoted unwinding of the stem and formation of 48S complexes with canonical toe-prints +15-17 nt downstream from the first AUG.

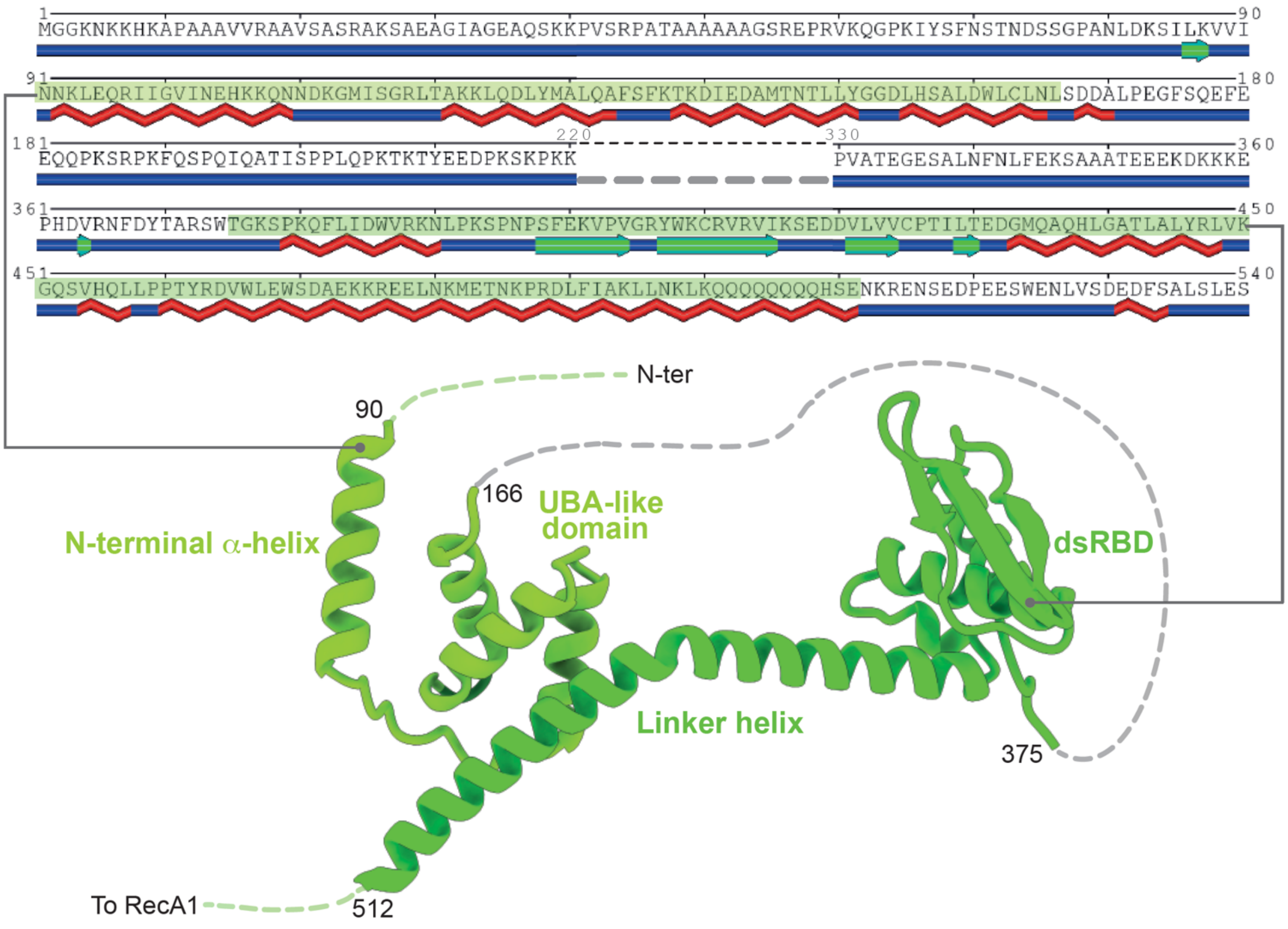

Figure S3. Sequence, the secondary and the tertiary structure elements of DHX29's NTR.

The upper panel represents secondary structure elements of DHX29's NTR with α-helices shown as red zigzag lines, β-strands as thick green arrows and coils as blue lines. Corresponding sequence and residue numbers are displayed above the secondary structure elements. A long unstructured coil between residues 220 and 330 was removed and marked by a dashed line for simplicity. The lower panel represents the tertiary structures corresponding to the secondary structure elements shown in the upper panel. Corresponding residues are highlighted in matching colors on the sequence above the secondary structure elements of the upper panel. The secondary structure elements of the upper panel were generated by POLYVIEW-2D web server (Porollo et al., 2004).

Porollo,A.A., Adamczak,R. and Meller,J. (2004) POLYVIEW: a flexible visualization tool for structural and functional annotations of proteins. Bioinformatics 20, 2460-2462.

Table S1. Summary of distances between of the sites of directed hydroxyl radical cleavages in 18S rRNA and corresponding modeled locations of Fe(II) tethered cysteines in DHX29's NTR.

Residue mutated to Cys in DHX29	Hydroxyl radical cleavage site in 18S rRNA		Distance	Relative intensity of cleavage
E418 (dsRBD)	helix 18	G601	24.4 Å	weak
		U602	22.6 Å	weak
	helix 33	A1278	26.1 Å	weak
		C1279	29.3 Å	weak
		A1280	33.3 Å	weak
		U1302	24.9 Å	weak
		U1303	29.1 Å	weak
		U1304	29.9 Å	weak
		C1305	31.2 Å	weak
		G1314	14.7 Å	strong
		U1315	15.5 Å	strong
		G1316	18.1 Å	strong
		G1317	17.9 Å	strong
		G1318	18.5 Å	strong
	helix 34	U1500	13.7 Å	strong
		U1501	11.8 Å	strong
		A1502	10.5 Å	strong
		G1503	8.4 Å	strong
		A1504	10.8 Å	strong
		U1505	12.5 Å	strong
		G1506	17.7 Å	strong
K393 (dsRBD)	helix 18	G601	18.2 Å	weak
		U602	15.5 Å	weak
	helix 33	G1316	19.1 Å	weak
		G1317	20.0 Å	weak
		G1318	21.4 Å	weak
	helix 34	U1500	6.5 Å	medium
		U1501	8.3 Å	medium
		A1502	11.0 Å	medium
		G1503	14.6 Å	medium
		A1504	11.5 Å	medium
		U1505	10.0 Å	medium
		G1506	17.5 Å	medium
G433 (dsRBD)	helix 31	A1237	42.0 Å	weak
		U1238	44.4 Å	weak
	helix 32	G1265	39.6 Å	weak
		G1266	41.9 Å	weak
		C1267	44.5 Å	weak
	helix 33	A1291	52.6 Å	weak
		U1292	50.1 Å	weak
		U1293	48.2 Å	weak
		U1302	49.2 Å	weak
		U1303	52.8 Å	weak
		U1304	58.9 Å	weak
		C1305	64.0 Å	weak

		G1314	42.4 Å	weak
		U1315	40.4 Å	weak
		G1316	41.4 Å	weak
		G1317	44.9 Å	weak
	helix 44	U1706	38.9 Å	medium
		A1707	37.5 Å	medium
		C1708	40.8 Å	medium
		G1808	48.1 Å	weak
		A1809	46.9 Å	weak
		G1810	45.1 Å	weak
S373 (immediately preceding dsRBD, flexible part)	helix 44	U1706	25.4 Å	strong
		A1707	24.1 Å	strong
		C1708	26.7 Å	strong
		U1709	31.8 Å	strong
		A1710	33.2 Å	strong
		G1718	38.7Å	weak
		A1719	37.8 Å	weak
		U1720	40.2 Å	weak
		G1721	43.0 Å	weak
		G1722	48.5 Å	weak
		G1808	27.9 Å	medium
		A1809	30.0 Å	medium
		G1810	32.1 Å	medium
S76 (preceding UBA-like domain, flexible part)	helix 16	C522	38.3 Å	medium
		A523	37.2 Å	medium
		G524	37.3 Å	strong
		G525	42.5 Å	strong
		G534	37.0 Å	strong
		G535	38.8 Å	strong
		G536	43.2 Å	strong
		G537	49.3 Å	medium
		A544	40.3 Å	strong
		A545	40.6 Å	strong
		U546	40.9 Å	strong
		U547	47.1 Å	medium
	helix 18	U599	38.6 Å	medium
		G600	36.7 Å	medium
		G601	40.1 Å	medium
		U602	44.1 Å	weak
		U627	44.0 Å	medium
		C628	46.0 Å	medium
		C629	51.7 Å	weak
	helix 33	C1275	12.3 Å	medium
		G1276	16.5 Å	medium
		G1277	21.5 Å	medium
		G1317	22.8 Å	medium
		G1318	22.1 Å	strong
		U1319	26.2 Å	medium
		G1320	29.1 Å	medium
Y151 (UBA-like domain)	helix 16	A519	20.2 Å	weak
		U520	17.3 Å	weak
		C539	38.9 Å	weak

		C540	35.8 Å	weak
	helix 17	G549	17.5 Å	medium
		A550	23.3 Å	medium
		A551	26.6 Å	weak
		C560	51.4 Å	weak
		U561	54.9 Å	weak
		A574	32.2 Å	weak
		C575	29.0 Å	medium
		G576	28.4 Å	medium
		A577	25.7 Å	weak
E511 (immediately following the polyQ stretch)	helix 16	A519	26.7 Å	weak
		U520	29.6 Å	weak
		C539	40.7 Å	weak
		C540	38.5 Å	weak
		U541	38.0 Å	weak
		G542	36.2 Å	weak
	helix 17	G549	13.6 Å	medium
		A550	15.2 Å	medium
		A551	14.8 Å	weak
		C560	29.9 Å	weak
		U561	33.8 Å	weak
		A574	11.4 Å	medium
		C575	10.6 Å	strong
		G576	12.3 Å	strong
		A577	15.5 Å	weak
